# Prevention of low back pain and its consequences among nurses’ aides in elderly care: a stepped-wedge multi-faceted cluster-randomized controlled trial

**DOI:** 10.1186/1471-2458-13-1088

**Published:** 2013-11-21

**Authors:** Charlotte Diana Nørregaard Rasmussen, Andreas Holtermann, Ole Steen Mortensen, Karen Søgaard, Marie Birk Jørgensen

**Affiliations:** 1 National Research Centre for the Working Environment, Lersø Parkallé 105, 2100 Copenhagen Ø, Denmark; 2 Department of Occupational Medicine, Køge Sygehus, Lykkebækvej 1, 4600 Køge, Denmark; 3 Institute of Sports Science and Clinical Biomechanics, University of Southern Denmark, Campusvej 55, 5230 Odense M, Denmark

**Keywords:** Participatory ergonomics, Cognitive behavioral training, Physical training, Musculoskeletal disorders, Workplace intervention, Health care workers

## Abstract

**Background:**

A high prevalence of low back pain has persisted over the years despite extensive primary prevention initiatives among nurses’ aides. Many single-faceted interventions addressing just one aspect of low back pain have been carried out at workplaces, but with low success rate. This may be due to the multi-factorial origin of low back pain. Participatory ergonomics, cognitive behavioral training and physical training have previously shown promising effects on prevention and rehabilitation of low back pain. Therefore, the main aim of this study is to examine whether a multi-faceted workplace intervention consisting of participatory ergonomics, physical training and cognitive behavioral training can prevent low back pain and its consequences among nurses’ aides. External resources for the participating workplace and a strong commitment from the management and the organization support the intervention.

**Methods/design:**

To overcome implementation barriers within usual randomized controlled trial designed workplace interventions, this study uses a stepped-wedge cluster-randomized controlled trial design with 4 groups. The intervention is delivered to the groups at random along four successive time periods three months apart. The intervention lasts three months and integrates participatory ergonomics, physical training and cognitive behavioral training tailored to the target group. Local physiotherapists and occupational therapists conduct the intervention after having received standardized training. Primary outcomes are low back pain and its consequences measured monthly by text messages up to three months after initiation of the intervention.

**Discussion:**

Intervention effectiveness trials for preventing low back pain and its consequences in workplaces with physically demanding work are few, primarily single-faceted, with strict adherence to a traditional randomized controlled trial design that may hamper implementation and compliance, and have mostly been unsuccessful. By using a stepped wedge design, and obtain high management commitment and support we intend to improve implementation and aim to establish the effectiveness of a multi-faceted intervention to prevent low back pain. This study will potentially provide knowledge of prevention of low back pain and its consequences among nurses’ aides. Results are expected to be published in 2015–2016.

**Trial registration:**

The study is registered as ISRCTN78113519.

## Background

A high prevalence of low back pain (LBP) among health care workers has persisted over the years despite extensive efforts in primary prevention in different countries. Health care workers typically cover a range of professions including among others doctors, nurses, occupational therapists, physiotherapists and nurses’ aides. For this study the main focus is on nurses’ aides working in elderly care. However, the work among health care workers engaged in care is similar and thus studies among health care workers in general will be referred to. The annual incidence of LBP among healthcare workers has been found to be 26% with a high yearly recurrence [1]. The one-year prevalence of LBP has therefore been found to be as high as 45% to 77% [2,3]. LBP may cause despair and discomfort for the individual, and is costly for the employers and the society [4]. Likewise, it can lead to several consequences such as impaired quality of life, work disability, sickness absence and early exit from the labor market [5,6]. Thus, initiatives to prevent and reduce LBP as well as the consequences of LBP are needed for this job group. Simultaneous prevention of both LBP and consequences may therefore be necessary for relieving the burden from LBP among health care workers.

Causes of the high prevalence, incidence and recurrence of LBP are multi-factorial [7]. However, the physical workload and frequent patient handling activities have in particular been reported to be a major contributing factor to LBP among health care workers [8-10]. Moreover, performing this type of work can lead to worsening of the pain condition among those already suffering from LBP [9]. Health care work is often performed by women with relatively low physical capacity [11], shown to be a risk factor for LBP among health care workers [12]. Additionally, health care workers without LBP who report high physical exertion have a high risk of developing chronic LBP [13]. Moreover, pain-related fear of movement (kinesiophobia) and fear avoidance beliefs has proven to be disabling and thus influential for sickness absence [14]. Among health care workers both with and without LBP, fear avoidance beliefs is related to future episodes of LBP [15]. Reducing the physical workload, physical exertion and improving fear avoidance beliefs and physical capacity may therefore prevent both LBP and its consequences among health care workers.

Many single-faceted interventions have been carried out at workplaces to prevent and reduce LBP, but with low success [16,17]. Single-faceted interventions only address one aspect of the multi-factorial origin of LBP [18,19] and may therefore not sufficiently target the origin or cause of LBP for each individual. A single-faceted intervention commonly used for prevention of LBP is to decrease the physical workload with ergonomic interventions such as introducing assistive lifting devices and training of correct lifting postures [16]. However, the effectiveness of such type of interventions in preventing LBP is not convincing [20,21]. In several reviews and process evaluations of ergonomic interventions, the importance of involving the participants in the planning and controlling a significant amount of their own work activities (participatory ergonomics) has been emphasized [22-25]. Participatory ergonomics is reported to be effective for preventing musculoskeletal disorders [22] and sickness absence [26].

Another single-faceted intervention used at the workplace to prevent LBP is physical training, but with limited evidence in reducing the prevalence of LBP [27,28]. Even though there is moderate documentation that physical training can reduce the severity of LBP and reduce sick leave due to LBP, it is far from clear what type, intensity, frequency and duration of training are optimal to prevent occurrence or recurrence of LBP and sick leave due to LBP in workers [7]. Strength training has shown effect on physical capacity and pain intensity in neck and shoulder among office workers [29], physical coordination training was able to improve recovery from chronic muscle pain among cleaners [30] and general physical activity has been shown to reduce duration of LBP [31]. Therefore, these types of physical training could also be an opportunity for effective prevention and reduction of LBP among health care workers.

The high risk of persistent and recurrent LBP among health care workers [1] calls for secondary prevention focusing on maintenance of functional activities despite pain, especially since their work is physically demanding. Thus with health care work being physically demanding, and the prevalence of pain being high, prevention of kinesiophobia and improving fear avoidance beliefs may be particularly important among health care workers. A means for this is cognitive behavioral therapy, shown to improve measures of coping such as catastrophising and pain-related fear of physical activity [32-34], musculoskeletal pain [35] and reduce days with sickness absence in a return-to-work program [36]. Cognitive behavioral therapy has been used among patients, but since this will be used as a preventive initiative among a non-patient group, we define the initiative as cognitive behavioral training (CBT) and not therapy. CBT could potentially serve as an effective secondary prevention strategy for workers with physically demanding work with high prevalence and reoccurrence of LBP.

A workplace intervention encompassing all employees must have a broad objective aiming at both prevention of LBP and its consequences [7] and an intervention must consist of several components involving different strategies. Thus a multi-faceted intervention consisting of participatory ergonomics, CBT and physical training should in theory be effective to reduce LBP and consequences among nurses’ aides. However, the effect of this combined initiative for prevention of LBP and consequences among nurses’ aides and delivered in a workplace setting still remains to be established.

For a workplace intervention to be effective, it needs to be implemented well in the organization [37]. However, multi-faceted workplace interventions are challenging to implement effectively because of their complexity and requirement of support from the organization and employees [38]. Especially among low-educated workers, studies regularly report problems with compliance and participation in workplace settings [39-42] and low effect [43,44]. The low participation and lack of implementation at the workplace can be due to lack of organizational resources to adopt intervention objectives, e.g. lack of support from supervisors [25,45] and from the organization [46]. Moreover the target population should find the intervention activities relevant, requiring a participating component to secure an effective implementation [37]. Therefore, a combination of various initiatives involving the participants and organization in the development, planning, performing and maintaining of the interventions [37] have been suggested as prerequisite for successful implementation [46,47]. The strict adherence to the randomized controlled trial (RCT) design can also be an implementation barrier, because it possesses a great risk of logistical issues and impaired organizational commitment, especially among the participants in the control group. A more feasible design for workplace studies may therefore be the stepped wedge design [48,49] introducing the intervention to all groups at the workplace but in a stepwise manner, where all groups get to serve as control groups until they step into the intervention.

**Figure 1:**
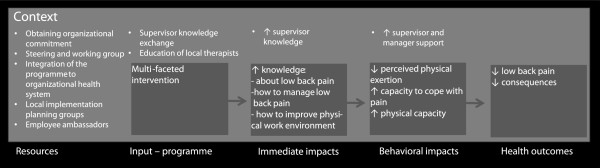
**The conceptual model of the study.** Formative effort was made to ensure organizational support for the intervention. The effort involved six overall steps: 1) obtaining organizational commitment, 2) steering and working group, 3) integration of the programme to organizational health system, 4) local implementation planning groups, 5) employee ambassador and, 6) supervisor support. The programme consists of education of local therapists to deliver the multi-faceted intervention, as well as ensuring supervisor support by having regular knowledge sharing meetings for the supervisors.

The main aim of this paper is to describe the design of a stepped-wedge multi-faceted cluster-randomized study among nurses’ aides with the aim of examining the effectiveness of an intervention consisting of integrated participatory ergonomics, physical training and cognitive behavioral training (CBT) with particular focus on participant and organizational involvement to prevent and reduce LBP and consequences of LBP. See Figure 1 for the conceptual model of the study.

More specifically, the study has three main hypotheses: 1) A 3-month multi-faceted intervention among nurses’ aides will reduce LBP compared to a control group receiving no intervention. 2) A 3-month multi-faceted intervention among nurses’ aides *pain-free at baseline* will prevent LBP compared to a control group receiving no intervention. 3) A 3-month multi-faceted intervention among nurses’ aides *with pain at baseline* will reduce consequences of LBP compared to a control group receiving no intervention.

## Methods

### Study design

**Figure 2:**
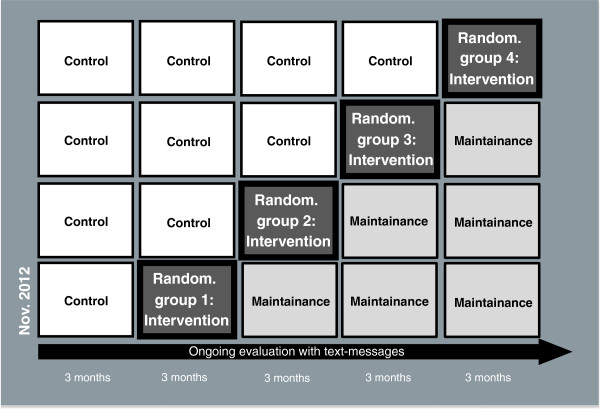
**The stepped-wedge design with four groups that are randomized to four successive time periods three months apart.** The data collection consists of monthly text messages. Moreover there will be further measurements with questionnaires every three months.

The study is described in accordance to the guidelines of the Consort Statement [50]. In clinical intervention research, the randomized controlled trial (RCT) is considered the gold standard. However, in workplace settings the introduction of control groups not receiving intervention can hamper implementation due to logistical issues and impaired organizational commitment [48,49]. Moreover, it is impossible to implement the intervention in many clusters simultaneously because of practical and logistical reasons. These difficulties can be overcome in the more feasible stepped wedge design [48,49] with gradual implementation of the intervention in different teams. Therefore, this study uses a stepped-wedge cluster-randomized controlled trial design with 4 groups (594 participants in total) (Figure 2). A stepped-wedge design is a type of crossover study in which clusters cross over from the control arm to the intervention arm at different time points [48,49]. Twenty-one clusters were formed based on working teams, and randomly assigned to four successive time periods three months apart and enrolled in the study in accordance with the stepped-wedge group order. The project began in November 2012 with baseline measurements and was followed by a 3 months burn-in period without intervention activities but with repetitive baseline measures. The intervention began in February 2013.

The study has been approved by the Danish Data Protection Agency and the Ethics Committee for the regional capital of Denmark (journal number H-4-2012-115) and will be conducted in accordance with the Helsinki declaration. The study is registered as ISRCTN78113519 in the current controlled trials register.

### Study population

Employees in elderly care (nursing homes and home care) in a larger municipality in Denmark participate in the study. The main employees in the elderly care in the municipality are nurses’ aides who are either social and health service (SHS) aides or helpers. In Denmark, SHS helpers have 14 months of training and are qualified for providing care of elderly people. SHS aides have an additional 6 months of training and are qualified for working in the eldercare sector, hospitals, and psychiatry. Eligible participants were nurses’ aides employed in elderly care more than 20 hours a week and being 18–65 years of age. For supporting implementation, participation was also offered to the kitchen and cleaning personnel as well as janitors belonging to the participating teams. Thus, the study population consists of low-educated service- and blue-collar workers in elderly care, but will be referred to as nurses’ aides. They were required to sign informed consent in order to participate. The exclusion criteria to the study were unwillingness to participate in the multi-faceted intervention, long term sick-listed or not being permanently employed.

### Recruitment of study population

The first contact with the municipality was established by contacting the director general of the health and care administration office in the municipality by email and telephone. A meeting was subsequently arranged between working environment consultants from the municipality, working environment representatives from the nurses’ aides as well as local union representatives. At the meeting, the aim, content and activities of the project were described in overall terms and the possibility of enrolment in the study was discussed.

After formal confirmation of collaboration the details about the recruitment of employees were settled. In this municipality, the administration of elderly care is divided into nine districts, representing geographically separated districts managed by separate district managers and counting approximately 4350 employees in total of which approximately 3000 are nurses’ aides. Each of the nine districts has between 3–6 nursing centers spread out geographically in the districts in which there are multiple working teams. The researchers then presented the study at a meeting for the district managers. Afterwards, they were given the opportunity to discuss the study with their employees and whether or not their district wanted to participate in the study. The district managers were encouraged to provide the reasons and motivations to participate in the study. The study was dimensioned to enroll 3–5 of the districts. Four of the nine districts consented to participate in the study.

In the fall 2012, all employees in the four districts were invited to a short information meeting of 30 minutes’ duration providing information about the project, and participants indicated their interest in participation. Prior to the information meeting, written information about the aim and activities was distributed to all employees in a short information brochure. Because of the team structure in the municipality, it was necessary to conduct several information meetings (>40) in order to reach as many of the employees as possible.

### Funding of the project

In Denmark, workplaces can apply for a grant through the national Prevention Fund (established in 2007), which covers the cost of implementation of workplace interventions in order to reduce musculoskeletal disorders, impaired health and work ability and sickness absence and thereby prevent exclusion from the labour market [51]. It was decided that the municipality should apply for a grant in order to cover some of the expenses of participating in the study. A project description was prepared for the application and the municipality was granted 6.8 million DKK (approximately 900.000 €) for the implementation of the intervention.

### Randomization

**Figure 3:**
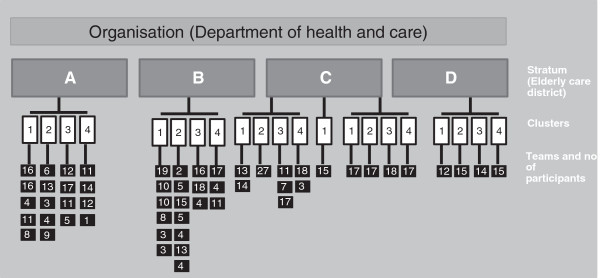
**Illustration of the cluster-randomization.** Strata were formed by each of the four districts and clusters were formed within each stratum. Small teams were merged to a cluster when located in geographical proximity.

The employees who volunteered for participation in the study were randomized to four successive time periods, three months apart in the stepped wedge design. Since the intervention is group-based, the randomization was performed across clusters based on working teams. Since the work-site locations of the participants are widely distributed over a large geographical area and the number of participants on the work-locations differs, a balanced cluster randomization was applied. Strata were formed by each of the four districts and clusters were formed within each stratum. To promote comparability between the clusters they were balanced on number of participants in each cluster to minimize imbalance over several strata. Therefore, we randomized the clusters according to their size with the four largest clusters randomized first. Due to logistics related to the intervention delivery, small teams were merged to a cluster when located in geographical proximity (Figure 3).

Strata were named alphabetically and clusters named consecutively within each stratum. One of the authors (CNR) stratified the participants into the strata and clusters, but was blinded to the succeeding randomization. All grouped clusters belonging to a specific stratum were drawn from a deck of cards with each color presenting a step from 1 to 4 in the study. Researchers blinded to the identity of the strata and clusters carried out the randomization. The participants do not receive information about which group they are randomized to until shortly before crossing over from control to intervention.

### Delivery of the intervention

Local therapists (3 occupational therapists and 3 physiotherapists) were trained to carry out the intervention activities. The training is guided by a written intervention protocol describing all intervention activities and 6 days of training and sparring sessions throughout the study period with CNR. The instructors will be delivering the intervention to the nurses’ aides. The intervention will be scheduled in the working time of the participants if feasible for the individual team. The working time spend on the activities by the employees will be compensated to the workplace by external funding obtained by the municipality.

### Organization of the implementation support system

Prior to the intervention, a formative effort was made to ensure organizational support for the intervention. The effort involved six overall steps.

1) **Obtaining organizational commitment**

Organizational commitment was obtained through broad information and informed commitment from the director general, the worker safety and work environment board and volunteer commitment from each of the district managers and their teams of nurses’ aides’ supervisors as described in the section "Recruitment of study population".

2) **Forming a steering group and a working group**

A steering committee was formed. The steering committee consists of a chairman (a manager of one of the four participating districts), the managers of each of the remaining three participating districts, a local project leader, two of the researchers, a local union representative (an employee), and a local working environment representative. A working group was also formed. The working group’s main task is to deliver material to the steering group for decision-making. The working group consists of a local project leader, the researchers and other relevant resources needed for specific tasks.

3) **Integration of the programme to the organizational health system**

Linkage to the higher administration as well as local occupational health system was arranged with training in the intervention’s aim and content. Target persons were the occupational working environment consultants, work environment representatives among the workers and top management of the administrative department. Furthermore, the intervention was designed to be delivered by local therapists to plant the knowledge and experience in the organization.

4) **Focus on supervisors**

The supervisors of the teams were trained in the interventions aim and content prior to the beginning of the study (in October 2012). Moreover they were required to form a support system with knowledge sharing meetings for supervisors in the three months when their team receives the intervention.

5) **Local implementation planning groups**

In each district local implementation planning groups are to be established with local work environment consultants, local employee’s representatives, supervisors of the teams, the project leader, the researcher and the therapist responsible for conducting the intervention. Approximately 8–10 weeks before each of the 4 groups enter the intervention they will meet and plan in details the logistics of implementation of the activities in the intervention for the participating teams, e.g. date and time for the different activities.

6) **Employee ambassadors**

In each team, an employee has been appointed a specific role as an ambassador and will be motivating the colleagues to participate in the study.

Throughout the intervention period, tailored information material about the process and role expectations will be sent to the ambassadors, supervisors, work environment consultants and managers.

### Intervention development and content

#### Development of the intervention

The intervention lasts 3 months and integrates participatory ergonomics, physical training and CBT tailored to the target group. The activities are specified and adjusted by a modified intervention mapping approach [52]. The intervention mapping facilitates participation and consultation of all participating stakeholders. The development of the intervention activities is based on four key points: 1) effectiveness; the activities should be effective 2) feasibility; the activities could be executed at the workplace during working hours, 3) motivation; workers should find the activities appealing and relevant and 4) evaluation; it should be possible to conduct a sound scientific evaluation meaning that the activities follows a standardized protocol [52].

**Figure 4:**
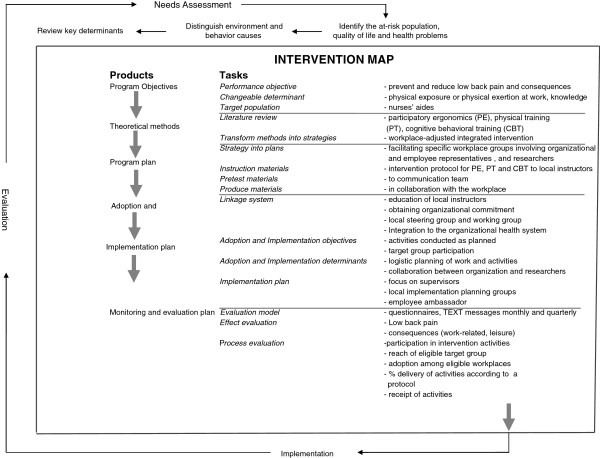
Overview of the intervention mapping procedure.

The tailoring of the intervention to the nurses’ aides started with a needs assessment by using existing registrations of the working environment in the municipality and searching relevant scientific literature. This helped specifying the objective of the intervention to both include prevention and reduction of LBP as well as preventing the consequences of LBP (e.g. work ability and sickness absence) among nurses’ aides (Figure 4).

To further tailor the intervention to the workers, the researchers performed a small qualitative evaluation of the work environment and occupational health services among the workers. The evaluation involved 1) observations of daily work life activities among the nurses’ aides as well as a short interview with the nurses’ aides and 2) observation of ergonomic classes held biannually in the municipality for the nurses’ aides. The data gathered in this phase indicated that activities should be carried out in proximity of their workplace and during their working time. The ergonomic classes focused on information of risk factors and training in manual patient handling and these factors were considered in the final content of the intervention activities, and documented in the intervention protocol. Finally, from the planning groups further adjustments will be made regarding the logistics of the activities, e.g. the one-hour physical training pr. week will be planned as once a week for one hour, twice a week for 30 minutes or whatever proves to be the most feasible for the individual team. The content of each of the activities will be described in further details in the following.

### Intervention

#### Participatory ergonomics

The overall aim of the participatory ergonomics in this study is to prevent physical exertion and pain through minimizing physical risk factors for LBP at work and reducing the work tasks perceived as physically demanding. Participatory ergonomics covers *"the involvement of the workers in planning and controlling significant amount of their own work activities, with sufficient knowledge and power to influence both processes and outcomes to achieve desirable goals"*[53]. The literature highlights participatory ergonomics as not being a unitary concept, but rather as an umbrella term covering a fairly broad range of ideas and practices [54]. In this study, inspiration from the framework suggested by Haines et al. [54] as well as the blueprint suggested by Wells et al., [55] was used in the development of the participatory ergonomics intervention. Therefore the participatory ergonomic process follows 6 steps: 1) identification of physically demanding work tasks, 2) analysis of physically demanding work tasks, 3) solution building, 4) prototype implementation, 5) evaluate prototype and 6) adopt solution. These steps will be carried out in two workshops of 3 hours and two follow-up meetings of one hour.

The participatory ergonomic process is initiated by a one-hour start-up meeting in each team. At this meeting, the ergonomic work group is formed. The ergonomic work group will consist of 5–7 employee team members and the instructor (a local therapist) will serve as the ergonomist. No supervisors are involved in the ergonomic work group. However they will be involved between workshops, as an employee (the ambassador) will present the decisions taken at the workshops for the supervisor. At the start-up meeting, all employees will have the opportunity to identify which work tasks they consider physically demanding and therefore a risk for development, maintenance and reoccurrence of pain. They are further encouraged to write down their suggestions and put it in an ergonomics mailbox situated at the workplace. The mailbox will be used throughout the intervention period and remain open for suggestions for the participatory ergonomic process from all the employees.

At the first workshop, the ergonomic work group will prioritize the identified physically demanding work tasks and analyze these. The results of this workshop should be 3–4 prioritized work tasks that should be 1) relevant (e.g. many workers perform the task or the task is done many times during a working day) and 2) be a significant risk factor for pain or it causes a high physical work exertion. At the second workshop, the ergonomic work group will come up with solutions to the prioritized work tasks and make an implementation plan. They are again asked to prioritize the solutions according to 1) efficiency (i.e. can this solution reduce pain or physical work exertion?) and 2) feasibility (i.e. is this solution likely to be implemented within the project period?). After the workshops, the solutions are to be implemented. At two follow-up meetings, the implementation of the solutions will be evaluated and possible adjustments made.

#### Physical training

The overall aim with the physical training in the study is to introduce different types of physical activities to the participants and motivate them to maintain the preferred physical training. The different types of physical training are chosen based on the evidence of efficiency as physical capacity building activities and their possible impact on preventing and reducing LBP. The physical training types are presented in 3 blocks: 1) body awareness and body postures, 2) strength and coordination training and 3) general physical activity. The physical training will be carried out each week for one hour with an instructor. It consists of twelve sessions with separate focus areas. The first four sessions will be introduction to physical training and the three different types of training. In the remaining sessions, the participants will be able to choose from the different types of training and develop their own training regime. During the sessions, different tasks will be incorporated that refers to ergonomic principles (e.g. manual handling of patients and good body postures) or cognitive behavioral principles (e.g. experience of acute vs. long term muscle discomfort/soreness/pain and training in relaxation techniques). Each training session starts with a warm up routine that will be the same for all twelve sessions and lasts approximately 15 minutes. The warm up routine consists of conditioning exercises involving large postural muscle groups as well as strengthening exercises for the abdomen and the lower back. The participants also receive a short brochure with description and illustrations of the exercises.

#### Cognitive behavioral training (CBT)

The CBT programme is a modified version of the program developed by Linton [56] and further developed for a working population by Jørgensen et al. [32]. The purpose of the programme is to reduce and prevent pain, and diminish negative effects of pain. All participants will be participating in two workshops of 3 hours. The workshops follow the same structure with a short lecture on the themes, problem-solving training and training of new skills (e.g. applied relaxation training). The first workshop will focus on improving the participants’ understanding of pain, the experience of pain, and the anticipation of pain by performing cognitive exercises on how physical activity may negatively or positively relate to pain. Another main focus will be on pain in relation to physical demanding work. The second workshop will focus on the ability to function and have a good life quality despite pain (i.e. pain coping, increasing health behavior, adapt skills to daily life). Moreover, the positive long-term effects from appropriate pain coping will be discussed [56]. Finally, the participants will make their own individual plan for using the new skills and each team will make a plan on how to implement the new skills in their working day.

#### Maintenance

At the end of the intervention, the teams will prepare for the maintenance phase. In the maintenance phase, the ergonomic work groups will transform into an ergonomics and health promotion group. That means, that they will continue using the skills obtained during the participatory ergonomics programme (identification, analyses, solution building, prototype implementation, evaluate prototype and adopt solution), but will now broaden their scope to also include health-related challenges and solutions within health promotion methods. Still, the groups’ work will be based on input and suggestions from all employees and they will develop action plans on how to continue and maintain the activities after the 3 months of intervention.

#### Knowledge sharing

Throughout the three months period of intervention, the supervisors of the participating teams will attend three one-hour meetings, one each month. The main topics of these knowledge sharing meetings will be barriers and facilitators for implementation of the intervention. At these meetings, the researcher will be participating and guide the discussions. Minutes from the meetings will be distributed to the involving supervisors afterwards.

### Efforts for high compliance

An important focus in this study is to maintain high compliance throughout the study. It is well known that maintaining participants in the study and high participation is a difficult process in intervention studies at the workplace [41]. Therefore, initiatives are taken to make participation more attractive and to minimize dropout. Firstly, the concepts of the project are participatory to ensure that the intervention is tailored to the specific needs of the participants facilitating ownership and motivation to participate in the study. We ensure that the entire organization is informed about the main features, purposes and processes of the project by having regular meetings. Written materials such as information letters, brochures and posters are distributed to the participants throughout the study period.

Furthermore, particularly for the stepped-wedge design, it is important that the same intervention content is delivered at each step (i.e. introduction of a new intervention group). To support this, the intervention mapping process was conducted to ensure the intervention was optimally tailored to the organization. Also, the intervention protocol was written with specific measureable criteria for delivery of the intervention. Finally, pre-developed expectation materials are delivered to all stakeholders of the intervention. Moreover information to support organizational level decisions about dissemination of the intervention content to other departments is withheld as long as possible until proper systematic evaluation can be conducted when all four intervention deliveries are finalized.

### Data collection

The data collection consists of text messages and questionnaires. At baseline the participants also received physiological health measures for describing the health of the population.

### Physiological health measures

To map the health of the employees and to motivate them to participate in the intervention, all participants were invited to physiological health measures at baseline. The height was measured with no shoes and in upright standing position (Seca 230). Body weight, body mass index (BMI) and body fat percentage was determined using bio impedance (TANITA BC-418). Resting blood pressure was measured on the left arm after at least 15 minutes sitting rest (Omron M3) [57]. Participants received individual feedback on the results from the health measures in regard to Danish and international guidelines [57,58]. All measurements were performed by trained clinical personnel (physiologists and physiotherapists).

### Evaluation

Analyzing the effects and processes of a complex intervention requires a comprehensive evaluation. In order to match the design and context of this project, the efficiency of the study will be evaluated. The study efficiency will be a result of both efficacy and implementation (Efficiency = Efficacy X Implementation) [59].

### Primary outcome measures

Because LBP is a fluctuating condition, which can be difficult to recall [60], monthly monitoring will be conducted by using mobile phone text messages. In addition to the frequent measuring of LBP, consequences (i.e. bothersomeness due to LBP) will also be measured monthly by text messages delivered by the SMS Track® system [61,62]. The setup of the software is designed for the study in close cooperation with researchers. Every month (on a Monday) the respondents receive an automated text message to their private mobile phone, which they are expected to answer by using a text message. LBP is measured as days with pain and intensity of pain in the lower back each month throughout the data collection period. The questions posed are "*During the previous four weeks, how many days have you had low back pain?"* and *"What was the highest intensity of your low back pain, with 0 being no pain and 10 being the worst imaginable pain?"* Consequences of pain will be measured by days with bothersomeness due to LBP each month throughout the data collection period. In a recent consensus report to standardize LBP measures, the description "limit your usual activities or change your daily activities" was suggested to measure the severity of pain [63]. Moreover, bothersomeness has shown to correlate well with quality of life [64], thus making a good measure for consequences of LBP. The question posed is *"How many days during the previous four weeks has your low back pain been bothersome (*i.e. *affected your daily activities or routines)?"*

### Secondary outcome measures

Self-reported sickness absence due to LBP is obtained monthly by text message [65]. Additionally, every three months the following will be measured by text messages: Work ability [66], Occupational lifting and carrying [67], Self-rated physical exertion [68], Self-rated muscle strength [69], fear avoidance beliefs [70] as well as support from management regarding support to handle musculoskeletal pain.

### Process evaluation (implementation)

An evaluation of the implementation will be performed, inspired by the framework by Steckler and Linnan [71] to gain insight into the extent to which the intervention is implemented as intended [59]. The implementation will be measured through dose delivered (the amount of intervention components actually delivered by the instructors) and dose received (employees participation in the activities) [71]. The delivery will be measured by asking the instructors to what extend they have followed the specific intervention activities in accordance to the defined criteria written in the intervention protocol (they will fill out a questionnaire after each activity). The dose will be measured by participation rate and by questionnaires to the participants after the intervention asking about their appraisal of the intervention [71].

### Sample size calculation

For sample size calculation we used the method described by Woertman et al. [72] for the stepped wedge design. The sample size is calculated for LBP intensity (numeric rating scale 0–10). Due to the fact that we were not able to find any relevant studies on workplace interventions measuring LBP intensity, we estimated variance from the study by Kovacs et al. on patients with non-specific low back pain [73]. The variance was set to 2.1. With an α of 0.05, a power of 0.8, and an intracluster correlation coefficient of 0.05, we calculated that we needed 65 participants in a stepped-wedge trial to allow analyses of LBP intensity.

The sample size calculation was based on a patient group meaning that all subjects will have pain and thereby it is possible to reduce pain in all subjects. The present study is a workplace intervention where all employees are invited to participate. Therefore we expect to enroll both participants with and without pain, meaning that we need a bigger sample size in order to detect a difference in LBP intensity. Workplace studies often have a high drop-out rate and could be expected to be up to 50%. When conducting a stepped wedge design, the intervention period is prolonged. This can be an extra risk factor for a high drop-out rate due to a high turn-over rate or due to "fatigue" relating to waiting for receiving the intervention. Moreover there is a greater risk for organizational changes happening at the workplace during the study period, meaning that we could lose entire clusters in the evaluation. Giving that we have a workplace willing to offer the intervention to all employees, we chose to randomize all 594 who wanted to participate and therefore should have sufficient power to detect an effect on LBP intensity even when taking all of the above concerns into consideration.

### Statistical analysis

Baseline characteristics will be described by questionnaires and the physiological health measures. Analyses regarding the effectiveness of the primary outcomes and secondary outcomes will be performed after three months of intervention by means of multilevel analyses suggested by Hussey & Hughes (linear mixed model (LMM) or generalized linear mixed models (GLMM)) [74]. Multilevel analyses take clustering of observations of workers within the same team into account, as well as repeated measurements within one participant [75]. The analyses will be conducted for the different measures of LBP; intensity, days and bothersomeness. Moreover, we will investigate whether the combined measure of the three measures of LBP will fulfill the criteria for a LBP index and investigate the effect on the LBP index.

All statistical analyses will be performed according to an intention-to-treat principle. In addition, per protocol analyses will be conducted for those groups that actually complete the intervention protocol. Further, in a subgroup analysis the effect on only the population of nurses’ aides will be evaluated.

### Handling of missing data and loss to follow up

Efforts to avoid missing data are conducted. Questionnaires are distributed personally to the participants through the instructors. If they are not present, the supervisors or ambassadors are advised to hand out the questionnaire to the participants later on and to encourage the participants to complete the questionnaire and to send it back in a stamped and addressed envelope. The text messages are sent on a Monday around lunchtime and a reminder is sent Wednesday if an answer has not been received. Supervisors are told to support text message replies during working hours and posters are placed at the workplace to remind participants to answer. If answers are still missing, we call the participant by phone to get their response.

Withdrawal from the intervention requires that participants personally take contact with the researchers. If they voluntarily give their reason(s) for discontinuing the intervention, these are registered. A flow diagram describing the dropout rate in intervention and control group will be conducted. Furthermore, analyses to identify possible different baseline characteristics between participants who drop out and participants who continue in the study will be conducted to describe the dropout population and the possible confounding introduced by that.

For the analyses to test hypothesis 1 and 3 in an intention-to-treat-manner, missing data are imputed as last observation carried forward based on the assumption that missing data are missing at random. For the analysis to test hypothesis 2, missing data of participants will automatically be censured at the first missing data measurement during follow-up – no matter the reason for the missing data (i.e. spot-like missing or complete drop-out).

## Discussion

This paper presents the design of a stepped-wedge cluster randomized controlled trial to investigate the effectiveness of a multi-faceted workplace intervention among nurses’ aides combining participatory ergonomics, physical training and CBT. To our knowledge, this is one of the most comprehensive intervention studies among nurses’ aides conducted at the workplace. Intervention effectiveness trials for preventing and reducing LBP and its consequences in workplaces with physically demanding work are few, and mostly unsuccessful. Part of the reason for negative results may be that most previous interventions have been carried out as single-faceted interventions that only address one aspect of the multi-factorial origin of LBP. Establishing the effectiveness of a multi-faceted intervention in workplace settings among high-risk groups such as nurses’ aides is therefore necessary.

Strict adherence to a traditional RCT design may hamper implementation and compliance. Therefore, by using a stepped-wedge design as a means to improve implementation and establishing the effectiveness of a multi-faceted intervention will provide knowledge of prevention of LBP and its consequences in workplace settings among high-risk groups such as nurses’ aides. Moreover having a participatory concept throughout the study and involving the organization and focus on supervisors, as important drivers of change will potentially promote implementation of the intervention.

Only a few studies have focused on the natural course (i.e. development without interventions) of LBP in detail [2] and no studies have focused on the course of LBP in detail after an intervention. An accurate description of a fluctuating condition such as LBP may require several points of measurement over time to describe the course in detail. Measuring pain can possess a risk of recall-bias [60,76]. By using frequent measures of LBP the study will contribute to a more detailed description of the natural course of LBP among workers and a possible reduction of LBP during the intervention. Many previous studies have not been able to show a significant reduction in pain among workers [16,17,20,21,30]. A reason for that could be that most studies in workplace settings combined participants with and without LBP in the analyses, and therefore make it impossible to draw separate conclusions about the effectiveness of the intervention in relation to prevention of LBP, reduction of LBP and prevention of consequences. In this study, we will measure both the entire population and stratify the population in workers with and without LBP. By analyzing the entire population, we will be able to catch fluctuations between LBP and no LBP at group basis and see if there is an effect on workplace level. By stratifying the population, we will be able to do separate analyses according to the hypotheses and get insight into the effect of the intervention on prevention of LBP as well as prevention of consequences of LBP and not just as a reduction of pain among the entire population.

This intervention may benefit employees as well as employers. If the intervention proves to be effective, the nurses’ aides will benefit from an improved health and working environment. These positive effects may potentially contribute to reduce sickness absence, prolonging working life and thereby be beneficial for the society as well. The results of this study will therefore provide comprehensive knowledge regarding prevention as well as reduction of LBP and its consequences among high-risk workers in a workplace setting.

### Strengths and limitations of the study

The cluster-randomized controlled trial design is a methodological strength, since it minimizes the risk of contamination between the intervention and reference group, and reduces the risk for bias. The use of a stepped-wedge design further strengthens the study by overcoming issues with impaired organizational commitment and disappointed participants in the control group since all participants will be offered the intervention, but still gives the opportunity for a sound scientific evaluation in an RCT design.

The systematic intervention mapping approach is a strong feature of the study. The experience and information obtained in the process of tailoring and implementing an intervention among nurses’ aides will be captured and hopefully, benefit both the present and future studies.

Another strength is the frequent monthly monitoring of pain and its consequences. This will potentially provide a more valid insight into the fluctuating LBP among workers with physically demanding work.

A limitation of the current study is that the intervention consists of several integrated components, which do not allow for separate evaluation of the effect of each individual component. As a consequence, eventual effectiveness of the intervention can only be attributed to the entire intervention. Since single-faceted interventions are considered insufficient to prevent LBP and consequences in workplace settings, it is particular important to measure the effect from the entire multi-faceted intervention. However, the evaluation of the implementation will focus on the entire intervention as well as on the separate activities and will therefore gain insight into the working mechanisms of the different activities of the intervention.

## Competing interests

The authors declare that they have no competing interests.

## Authors’ contributions

CNR, MBJ, AH, KS and OSM participated in the discussion of the conceptual design of the study and wrote the initial protocol as well as the application for funding. CNR was responsible for drafting the paper, writing the trial registration and application for the ethical committee. All authors have read and commented on the draft version as well as approved the final version of the manuscript.

## Pre-publication history

The pre-publication history for this paper can be accessed here:

http://www.biomedcentral.com/1471-2458/13/1088/prepub
